# Sudden Vision Loss as the First Sign of Sepsis—Bilateral Endogenous Endophthalmitis of Uncommon *Capnocytophaga canimorsus* Etiology

**DOI:** 10.3390/medicina60060896

**Published:** 2024-05-29

**Authors:** Małgorzata Łątkowska, Małgorzata Gajdzis, Anna Turno-Kręcicka, Julia Kręcicka, Małgorzata Mimier-Janczak, Izabela Górczyńska, Radosław Kaczmarek

**Affiliations:** 1 Department of Ophthalmology, University Clinical Hospital, 50-556 Wrocław, Poland; 2 Department of Ophthalmology, Wrocław Medical University, 50-556 Wrocław, Poland

**Keywords:** endogenous endophthalmitis, endophthalmitis, *Capnocytophaga canimorsus*

## Abstract

We present a case of bilateral endogenous endophthalmitis with an extremely rare etiology of *Capnocytophaga canimorsus*. A 42-year-old asplenic patient with bilateral deterioration of visual acuity presented to the Emergency Department. The sudden deterioration of visual acuity, which prompted the patient to visit the ophthalmologist, was the first sign of the onset of sepsis. The physicians’ attention, in addition to poor visual acuity and intense inflammation on ophthalmologic examination, was drawn to the reported flu-like symptoms. They were accompanied by high C-reactive protein results and abnormalities in echocardiography. A blood culture isolated the bacterium *Capnocytophaga canimorsus.* Immunocompromised patients are particularly susceptible to *C. canimorsus* infection. Endophthalmitis of this etiology has a very aggressive course, both ophthalmic and systemic. Therefore, quick diagnosis and initiation of adequate therapy are crucial.

## 1. Introduction

*Capnocytophaga canimorsus* is a rare causative pathogen of intraocular infections [1,2]. So far only two cases of bilateral endogenous endophthalmitis of this origin have been described [3,4]. We present the first, to our knowledge, documented case, in which, in addition to inflammatory lesions on the fundus, Roth’s spots were seen.

Endophthalmitis is a vision-threatening ophthalmological emergency. The endogenous form involves pathogen hematogenous spread from the primary focus of the infection. The etiology of *C. canimorsus* is extremely dangerous with a high mortality rate in sepsis [5,6,7]. Due to rapidly increasing intraocular inflammation and an initially unclear cause, the prognosis of visual acuity is unfavorable [1,2].

## 2. Case Description

**Figure 1:**
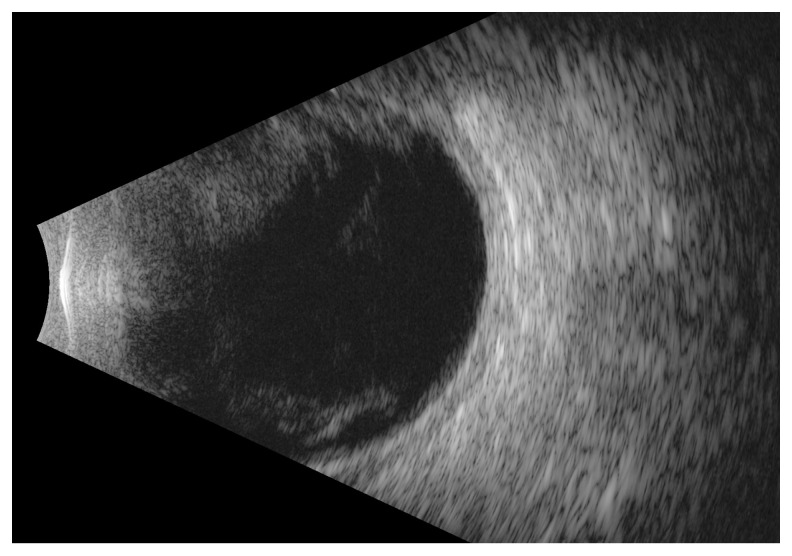
B-scan ultrasound of the left eye shows hyperechoic material in the vitreous chamber on admission.

A 42-year-old man with no history of ophthalmic diseases was transferred from another hospital to the Emergency Department (ED) due to significant deterioration of vision in both eyes. At the ophthalmological examination in the ED, the best corrected visual acuity (BCVA) of the right and left eye was finger counting at 50 cm and 20 cm, respectively. Slit-lamp examination demonstrated bilaterally the following: severe irritation, keratic precipitates on the cornea, significant hypopyon in the anterior chamber, posterior synechiae, and fibrin membrane on the lens. Due to intense inflammation seen in the anterior segment of the eyes, the fundus was not visible. The B-scan ultrasonography (USG) showed bilateral optic disc edema and hyperechoic material in the vitreous chambers, mainly in the left eye (Figure 1).

The patient had a fever of 38 °C and reported flu-like symptoms (fatigue, muscle aches) with onset a few days earlier. Non-ophthalmic symptoms raised clinical suspicion. He also had an increased risk of infection as he had undergone a splenectomy in the past. Complete blood count (CBC), biochemical tests of blood and urine, HLA antigens, and additional tests for viral and atypical infections were taken. Blood and urine cultures were also performed.

**Figure 2:**
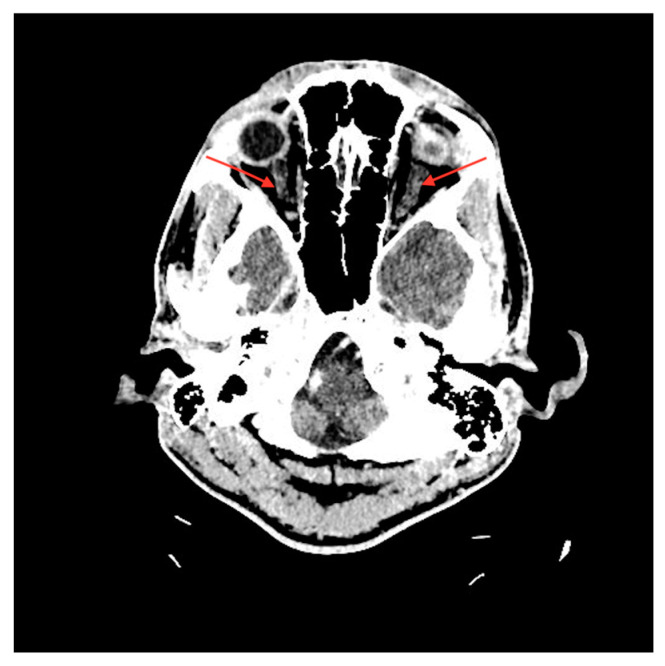
CT scan shows symmetrical thickening of optic nerve sheaths (red arrows).

A urine test was positive for amphetamine and marijuana. Laboratory tests revealed high levels of T troponin with an increase up to 2709 pg/mL (reference range: 0–34.2), thrombocytopenia of 36 × 10^3^/μL (reference range: 140–440 × 10^3^), and severely elevated inflammatory parameters including C-reactive protein of 249 mg/l (reference range: 0–5), indicating a suspicion of sepsis. Echocardiography revealed left ventricular hypokinesis and mildly reduced ejection fraction. Cardiologists, after additional tests including coronary computed tomography angiography (CCTA), ruled out an acute myocardial infarction and endocarditis. Myocardial damage was attributed as a secondary sign of ongoing infection. In addition, the thickening of the optic nerve sheaths on computed tomography (CT) was described (Figure 2). A neurologist, after neurological examination along with a head CT, ruled out neuroinfection as the cause of the presented symptoms. Due to the patient’s deteriorating condition, he was admitted to the Department of Internal Medicine.

**Figure 3:**
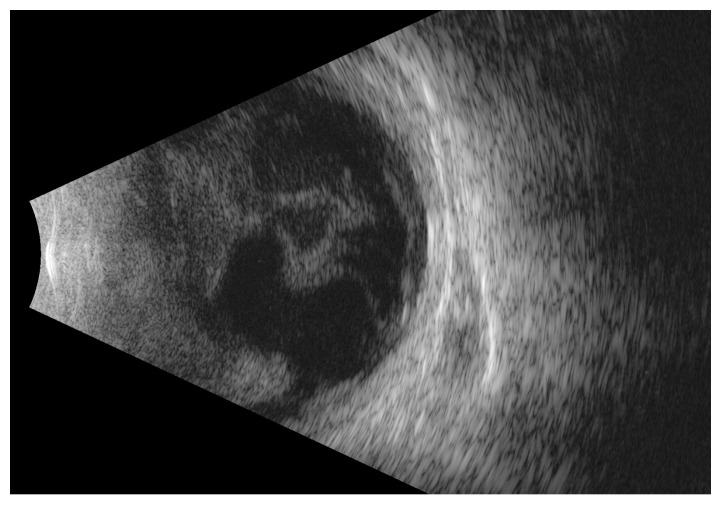
B-scan ultrasound of the left eye shows progression with a rapid increase in hyperechoic material in the vitreous chamber after 24 h.

Intravenous broad-spectrum antibiotic therapy, specifically ceftriaxone and vancomycin, was instituted. Within 24 h, there was a progressive decline of vision to hand motion (HM) in front of the eyes. Also, the vitreous exudate, visible on USG scans, increased rapidly (Figure 3). Because of bilateral endophthalmitis with an initially unknown starting point, pars plana vitrectomy (ppV) was performed consecutively on both eyes. The anterior chamber washout was also performed, and the pupil was relieved from posterior synechiae. Samples were taken from the inflamed vitreous for culture. After the removal of the vitreous body, inflammatory and necrotic lesions on the retina with inflammation of the vessel walls were visualized. More intense changes were observed in the right eye. With each day of antibiotic therapy, the patient’s condition improved. A blood culture resulted in the isolation of *Capnocytophaga canimorsus*—a bacterium normally found in the oral flora of dogs and cats [1]. The moment of infection most likely occurred about a week earlier, when the patient suffered a dog bite—traces of healing wounds were still visible on the fingers of the right hand.

**Figure 4:**
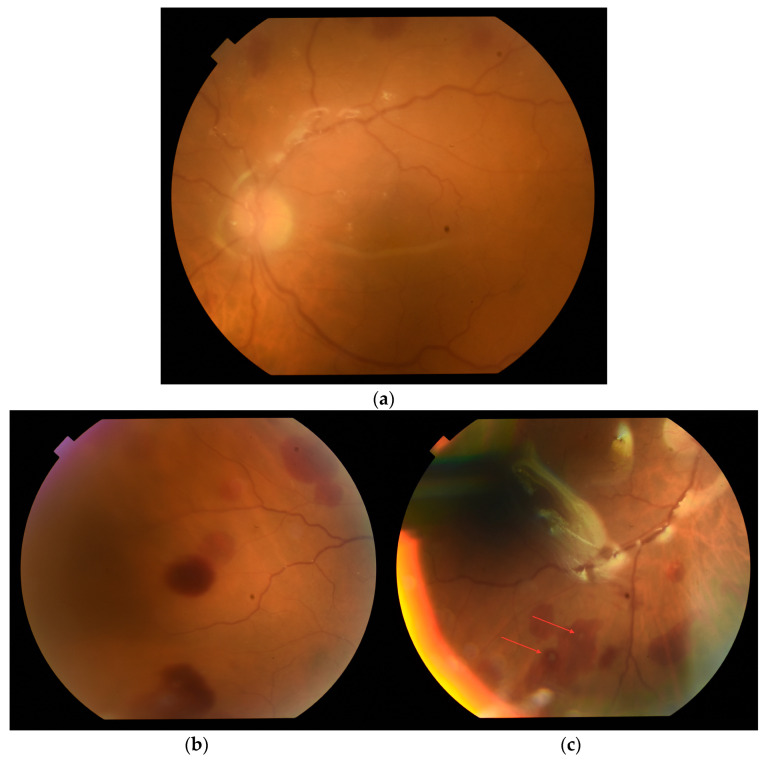
Fundus photography shows (**a**) the posterior pole of the right eye image at the first follow-up after vitrectomy. The vitreous chamber was filled with an endotamponade of silicone oil. (**b**) Intraretinal hemorrhages located outside of the major temporal vascular arcades. (**c**) Roth’s spots (oval hemorrhages with a central white spot) on the periphery of the retina (red arrows). They are associated with multiple systemic diseases—bacteremia (mostly secondary to infective endocarditis), leukemia, or hypertensive retinopathy.

After the surgery, insight into the fundus improved (Figure 4a). In the postoperative follow-up, retinal inflammatory changes with intraretinal hemorrhages were seen (Figure 4b). Roth’s spots could be noticed in the peripheral retina. (Figure 4c).

At the recent check-up, one week after surgery, the BCVA of the better eye was 20/100. Due to a significant improvement in the patient’s condition and normal values of inflammatory parameters, the patient was discharged with further ophthalmological care in the outpatient clinic.

## 3. Discussion

Sudden loss of vision is an emergency and a challenge in ophthalmologic management—it is only a symptom, which can be associated with multiple conditions. Due to the variety of possible causes, a multidisciplinary approach is often the key to appropriate diagnosis. Neurovascular problems such as neuropathy of the optic nerve due to ischemia or cerebral stroke with blockage of the blood flow to the occipital lobe are a common cause. It may be crucial to perform a neurological examination and brain imaging to identify possible ischemic lesions. In the differential diagnosis, retinal vessel occlusion, retinal detachment, vitreous hemorrhage, ocular injury, infections, or underlying autoimmune diseases with ocular symptoms should be considered. Acute vision loss can also occur as a result of the toxic effects of drugs and metabolic changes [8,9,10].

In the patient described above, uveitis could also be diagnosed due to severe irritation, inflammation of the anterior chamber, and exudate in the vitreous chamber. However, non-ophthalmic manifestations, i.e., elevated inflammatory laboratory markers, elevated body temperature, and immunosuppression due to asplenia, evoke a suspicion of a systemic infectious background.

Endogenous endophthalmitis occurs when microorganisms spread by the blood-borne route and enter the inner spaces of the eye through the blood–eye barrier [11]. It is usually a difficult disease for an ophthalmologist to manage due to the lack of identifiable systemic sources, aggressive progression, and poor clinical prognosis. The endogenous form accounts for only 2–8% of all endophthalmitis cases; the others are those with an exogenous cause. [12].

The worldwide incidence of endogenous endophthalmitis is on the rise. However, difficulties in the ambiguous clinical picture cause delayed diagnosis. The patient may report symptoms of underlying systemic infection such as fever or fatigue in addition to ocular complaints [13]. The ophthalmologic examination reveals decreased blurred vision and red eye [14].

A significant factor in the development of endogenous endophthalmitis in the case of the described patient may have been a history of splenectomy as well as drug abuse. Yet substances administered intravenously were not detected in his urine. Injection drug use (IDU)-associated endogenous endophthalmitis is more common in young men, who do not suffer from comorbidities [15]. Many studies associate the immunosuppression condition with a higher risk of developing endogenous endophthalmitis. However, there is no uniformity on this issue. Among the predisposing factors are diabetes mellitus, organ transplantation, systemic malignancy, human immunodeficiency virus (HIV) infection, or systemic immunomodulatory therapy. Coexisting conditions causing immunosuppression are often associated with a fungal etiology [16,17]. In contrast, there are reports describing that immunosuppression does not significantly increase the risk of developing bacterial endogenous endophthalmitis (BEE). This hypothesis assumes that transplant patients are under constant medical surveillance and may have antibiotic therapy implemented earlier [18].

Patients with bacteremia extremely rarely (0.05%) develop BEE. And only in half of the cases can the infectious agent be identified. The most common pathogens are Streptococcus, Staphylococcus, or Serratia species. Endocarditis is the most frequent disease among other comorbidities which account for BEE [14,18]. Men with bacteriemia without BEE appear to have a higher mortality rate. This may be related to the onset of distressing ophthalmic symptoms, which prompt patients to see a physician sooner.

In the patient described above, *C. canimorsus* was isolated from the blood. Members of the genus Capnocytophaga are slow-growing, Gram-negative bacilli that rarely cause ophthalmic infections such as keratitis, conjunctivitis, or blepharitis. While some species of Capnocytophaga (*C. ochracea*, *C. gingivalis*) belong to the normal human oral bacterial flora, *C. canimorsus* is usually not found in humans. It forms part of the gingival flora of animals, mostly dogs and cats. The most common way for a human to become infected is through a dog bite, rarely through a cat scratch or close contact with animals. Unlike other Capnocytophaga species, *C. canimorsus* infection can manifest with several systemic disorders, such as sepsis, meningitis, cellulitis, and endocarditis [1,19].

To date, single cases of bilateral endogenous endophthalmitis of this etiology have been described. Immunosuppression, and especially asplenia, as predisposing factors are emphasized [19,20]. Although *C. canimorsus* infections most often occur in immunocompromised individuals, they may happen in immunocompetent patients as well [3].

The clinical course of *C. canimorsus* endogenous endophthalmitis can be very aggressive. It begins with a rapid decrease in BCVA. Slit lamp examination shows significant irritation, cornea with Descemet membrane folds, hypopyon in the anterior chamber, fibrin deposits on the lens, and dense vitritis [2,3]. Interestingly, once the fundus is visualized, choriocapillary infiltration may be revealed [3]. Roth’s spots, which appeared on the retina of the described case, have not yet been described in the clinical picture of this disease entity. However, they were noted in endogenous endophthalmitis of a different etiology [21]. The extraocular prodromal symptoms, such as malaise or fever, may escalate quickly, leading to sepsis with high inflammatory parameters and organ failure. Sepsis of *C. canimorsus* etiology may be fulminant and fatal [5,6,7].

The accurate therapeutic pathway is difficult to specify, given the small number of cases of endophthalmitis with *C. canimorsus* etiology. Treatment is initiated empirically after cultures are taken. Sometimes, it is not possible to detect the infectious agent from blood cultures, and thus diagnostic ppV is necessary [2]. Treatment includes intravenous antibiotic therapy and intravitreal injections [3,4]. Intravenous ceftriaxone (1–2 × 2000 mg daily) is usually administered, less often clindamycin (3 × 300 mg) or ciprofloxacin (2 × 400 mg)—it is consistent with *C. canimorsus* antibiotic susceptibility [2,3,22]. Intravitreal vancomycin (1 mg/0.1 mL) in combination with ceftazidime (2.25 mg/0.1 mL), which are considered the intravitreal treatment of choice for successful management of endophthalmitis as they have a broad-spectrum effect, are most often used. Since the disease can take a very violent course or not improve after conservative treatment, there is a tendency to perform complete ppV [2,3]. Also, in complications of *C. canimorsus* infection such as retinal detachment or proliferative vitreoretinopathy, ppV is undertaken [1]. After completing the treatment, patients usually experience improvement in visual acuity, but it varies significantly depending on the severity of the disease [2,3,4].

Initially, given the ophthalmic clinical picture resembling uveitis and high CRP, *C. canimorsus* endophthalmitis can be mistaken for autoimmune inflammation, causing a delay in correct diagnosis and inadequate treatment [3]. Diagnostic difficulties in such a rapidly and severely progressive disease threaten a favorable prognosis for satisfactory visual recovery.

## 4. Conclusions

Due to the potential risk of serious infection, ophthalmologists should be mindful of the occurrence of *C. canimorsus* infection, especially in immunocompromised patients. Considering both the sight- and life-threatening complications of *C. canimorsus* endophthalmitis, early recognition and prompt implementation of appropriate treatment are crucial for positive clinical outcomes.

## Data Availability

No new data were created or analyzed in this study.
